# Monthly Summary

**Published:** 1886-11

**Authors:** 


### Monthly Summary.

---

IMPROVED METHOD OF OPERATING FOR CLEFT PALATE.—A correspondent writes to *The Lancet*, concerning what he considers a great improvement in the operation for cleft palate. Hitherto great difficulty and not a little danger have arisen from hemorrhage during the operation, necessitating frequent and very skillful assistance, periodical discontinuation of the anæsthetic, and distinct intervals in the performance of the operation. In addition to these, other and minor troubles are experienced. All these difficulties may be avoided, and the operations rendered perfectly safe and easy, by the simple process of inversion as apply to the head only. This can easily be attained by bringing the patient's shoulders well up to the end of the operating-table, and allowing the head to hang over the edge in the fully extended position. In this position the roof of the mouth would be horizontal or slightly inclined downward towards the operator, who should stand at the head of his patient. The anæsthetic is given through the nose by a small tube, and is quite out of the way of the surgeon. Only one assistant is required, who should stand to the left of the operator. In paring the edges, no change of hands is required, but the corresponding hand should be used in elevating the tissues of the hard palate, and in passing the sutures. Under these circumstances no blood can enter the larynx or œsophagus, the palate remains unobscured by blood, and whatever hemorrhage occurs finds its way into the nasal cavities, and at the conclusion of the operation may be emptied by simply turning the patient's head to one side.

---

SWALLOWING A SET OF ARTIFICIAL TEETH.—A remarkable surgical operation was performed at the Massachusetts General Hospital by Dr. Maurice H. Richardson, of Boston. About a year ago John McCarthy swallowed a set of artificial teeth. The passage of food to the stomach was almost wholly prevented, the patient grew emaciated and weak and it became evident that unless relief was had he must soon die. Dr. Richardson made a transverse cut in the left side of the abdomen, through which the man's stomach was drawn out and then cut open, when by the insertion of his arm to the elbow Dr. Richardson was able to reach and remove the teeth. The internal opening was then closed with fine silk and the stomach replaced, the external cut being closed with stitches. The whole operation was completed in forty-five minutes. The patient is doing well and his complete recovery is now considered little less than certain.

